# TORCH agents in women of fertile age: towards prevention of congenital infections, Italy, 2019 to 2020

**DOI:** 10.2807/1560-7917.ES.2026.31.4.2500135

**Published:** 2026-01-29

**Authors:** Claudia Pavia, Maurizio Zavattoni, Luigia Scudeller, Ambra Vola, Pierangelo Clerici, Massimo De Paschale, Francesca Genco, Cristina Giraldi, Francesca Greco, Valeria Meroni, Tiziana Lazzarotto, on behalf of the Torch Study Group

**Affiliations:** 1 GliTraVe, Vertically transmitted infections study group; Italian Association of Clinical Microbiologist (AMCLI), Italy; 2 SC Microbiology, Legnano Hospital, Aziende Socio Sanitarie Territoriali Ovest Milanese, Legnano, Italy; 3 SSD Grant Office, TTO and Scientific Documentation Unit, Scientific Direction, Fondazione IRCCS Policlinico San Matteo, Pavia, Italy; 4 Microbiology and Virology unit Diagnostic medicine Department, Fondazione IRCCS Policlinico San Matteo, Pavia, Italy; 5 Laboratory of Clinical Pathology and Microbiology, ASST Lodi, Lodi, Italy; 6 Microbiology and Virology Unit, AO Annunziata, Cosenza, Italy; 7 Department of Molecular Medicine, University of Pavia, Pavia, Italy; 8 Microbiology Unit, IRCCS Azienda Ospedaliero-Universitaria di Bologna, Bologna, Italy; 9 Microbiology Unit, DIMEC, Alma Mater Studiorum Università di Bologna, Bologna, Italy; 10 The members of the Torch Study Group are listed under Acknowledgements

**Keywords:** TORCH, Seroprevalence, Congenital infections

## Abstract

**BACKGROUND:**

Microbiological surveillance during pregnancy is important for better neonatal outcomes.

**AIM:**

We aimed to assess IgG and IgM antibodies against *Toxoplasma gondii*, parvovirus B19, *Treponema pallidum*, rubella virus and cytomegalovirus in women of fertile age (16–45 years) in Italy and investigate factors associated with the presence of antibodies.

**METHODS:**

We collected data from clinical microbiology laboratories on test results for IgG and IgM antibodies against *T. gondii*, parvovirus B19, *T. pallidum*, rubella virus and cytomegalovirus between 1 July 2019 and 30 June 2020. Serological tests, like IgG avidity for *T. gondii* and cytomegalovirus, non-treponemal tests for *T. pallidum* and molecular tests for parvovirus B19 and rubella virus were considered as confirmatory tests for acute infections. We investigated associations between presence of antibodies with age, nationality and geographic area of residence.

**RESULTS:**

Thirty-two laboratories submitted test results on 342,095 women. The overall weighted proportion of IgG antibodies was 13,700 of 111,580 (13%; 95% confidence interval (CI): 12–14) for women tested for *T. gondii*, 3,298 of 5,138 (65%; 95% CI: 60–69) for parvovirus B19, 63,828 of 69,865 (87%; 95% CI: 85–88) for rubella virus and 45,558 of 71,013 (66%; 95% CI: 64–68) for cytomegalovirus. For *T. pallidum*, 889 of 81,401 (1%; 95% CI: 1–1) of treponemal tests were positive. Overall, we estimated 530 acute infections with *T. gondii*, 33 with parvovirus and 449 with cytomegalovirus.

**CONCLUSION:**

These findings underline the need for screening for congenital infections in fertile women.

**Key public health message**

**What did you want to address in this study and why?**

Infections during pregnancy can lead to severe consequences to the fetus and newborn. We studied protection against so called TORCH pathogens including *Toxoplasma gondii*, parvovirus B19, *Treponema pallidum,* rubella virus and cytomegalovirus but excluding herpes simplex virus, among women of childbearing age (16–45 years) in Italy and the estimated number of new infections in one year, by age, geographic area and nationality.

**What have we learnt from this study?**

Immunity to these infections varied from 1% for *T. pallidum*, the causative agent of syphilis, to 87% for rubella virus, the latter is the only one of these microbes for which a vaccine is available. Our results also showed regional and demographic differences in seropositivity rates, underscoring the need for tailored screening and prevention initiatives.

**What are the implications of your findings for public health?**

Our study results stress the importance of maternal surveillance programmes in the prevention of neonatal infections. The results highlight that fertile women might benefit from specific advice on preventive measures and closer clinical follow-up.

## Introduction

The acronym TORCH is used to indicate infections caused by *Toxoplasma gondii* (T), other pathogens (O) referring for instance to the causative agent of syphilis, *Treponema pallidum*, and to parvovirus B19, rubella virus (R), cytomegalovirus (C) and herpes simplex virus (H) [1]. In healthy individuals, these infections are generally mild, but during pregnancy, these pathogens can cross the placenta and, when transmitted to the fetus, may lead to severe consequences (e.g. spontaneous abortion, stillbirth, blindness, deafness, neurodevelopment deficit, postnatal sequelae) [2]. Exposure to TORCH pathogens is affected by environmental conditions such as climate, season, vaccination coverage, cultural dietary practices, as well as by socioeconomic factors, maternal age, rate of preschool attendance by siblings and vaccine adherence [3-6]. Vaccination is currently available only against rubella virus, and in Italy it is offered within the child immunisation programme [7-9].

Toxoplasmosis is a zoonosis caused by the parasite *Toxoplasma gondii,* usually acquired via ingestion of contaminated food or water and rarely via transplanted organs [2]. Prenatal serological screening is essential for identifying women at risk and for appropriate actions (e.g. monthly screening, dietary counselling and treatment) to prevent transmission to the fetus [10].

Parvovirus B19 circulates in all areas of the world with seasonal outbreaks occurring every 3–5 years [2]. In European countries, seroprevalence in pregnant women varies from 55% to 74%. The incidence of parvovirus infection in pregnancy is approximately 1-2%, varying by season or during outbreaks. Fetal anaemia and hydrops are observed after maternal infection during pregnancy [11].

Syphilis causes more than 350,000 adverse pregnancy outcomes each year worldwide (over half as stillbirths or neonatal deaths) [7]. The recommended preventive actions include (i) strengthening surveillance, with programme monitoring and progress evaluation; (ii) prevention of sexually transmitted infections (STI); (iii) early diagnosis of STIs; (iv) patient and partner management and (v) approaches to reach the most vulnerable population groups. Targets and milestones of the global health strategies on STIs for 2022–2030 by World Health Organization (WHO) include 90% reduction of the global incidence of syphilis from 2018 and ≤ 50 cases of congenital syphilis per 100,000 live births in 80% of countries [12].

The acute illness caused by rubella virus infection is usually mild and characterised by fever and rash. If contracted in early pregnancy, it can result in congenital rubella syndrome of the fetus, with severe consequences such as cataracts, sensorineural hearing impairment, congenital heart disease, jaundice, purpura, hepatosplenomegaly and microcephaly [2].

Cytomegalovirus (CMV) is a major cause of congenital infections globally [2]. Infections in pregnancy can be primary (contracted during pregnancy) or non-primary (reinfection with other viral strains or, more frequently, reactivation). Primary maternal cytomegalovirus infection has a high risk of in-utero transmission. Congenital infection can present with petechiae, hepatosplenomegaly, jaundice, microcephaly, thrombocytopenia and hearing loss. In some states of the United States (US) for example, targeted screening of newborns for CMV is implemented [13].

During pregnancy, early recognition of TORCH infections for which treatment is available is important to prevent fetal infection and adverse fetal and neonatal outcomes. Also, where no specific treatment is available, a correct fetal monitoring, amniocentesis and fetal management plan should be offered. Therefore, national maternal and child healthcare programmes require accurate data on the incidence and prevalence of these infections.

Presence of IgG and absence of IgM antibodies against a specific pathogen are considered a sign of a previous infection or vaccination, while negative IgG and IgM results indicate a non-immune, susceptible person [14]. Presence of IgM and IgG antibodies indicate a recent or acute infection. Additional serological or molecular tests are necessary to better evaluate the risk of transmission. Thus, IgG and IgM serosurveys can be used to measure immunity and vaccination levels.

A correct microbiological surveillance, through the evaluation of serological status, monitoring and counselling of seronegative women of fertile age and during pregnancy, results in better neonatal outcomes, and knowledge of prevalence of these infections in the population is a prerequisite for designing and maintaining serological screening programmes [2]. Currently, only local seroprevalence data about TORCH pathogens are available in Italy [15].

We aimed to estimate the presence and distribution of antibodies against *T. gondii*, parvovirus B, *T. pallidum*, rubella virus and cytomegalovirus in fertile women to help guide public health initiatives aimed at reducing the overall burden of maternal–fetal infectious diseases. We did not include herpes infections as no serological screening is performed in Italy and infections are diagnosed only in symptomatic cases by molecular tests.

## Methods

### Microbiological surveillance of pregnant women

In Italy, serological screening in first trimester and monthly re-testing of non-immune pregnant women is not mandatory but recommended and offered free of charge for *T. gondii*, rubella virus and *T. pallidum*, and widely performed [10,16]. At the time of data collection of our study, testing for CMV and parvovirus B19 during pregnancy was not recommended but parvovirus B19 is tested when the pregnant person or the fetus shows signs of parvovirus infection.

### Study design

We invited all Italian clinical microbiology laboratories included on the Italian Association of Clinical Microbiologist (AMCLI) mailing list to participate in a multicentre study on a voluntary basis. The laboratories were asked to submit pooled results of routine serological testing of all women aged 16–45 years, irrespective of pregnancy status, for *T. gondii*, parvovirus B19, *T. pallidum*, rubella virus and CMV between 1 July 2019 and 30 June 2020. Data were pseudonymised and then pooled by the laboratory prior to submission.

### Data collection

The laboratories were asked to submit test results by age (16–25, 26–35, 36–45 years) and nationality (Italian or other nationality). The geographic region the laboratory covered was grouped into three areas: northern Italy (Emilia Romagna, Friuli Venezia Giulia, Liguria, Lombardy, Piedmont, P.A. Bolzano, Aosta Valley, Veneto), central Italy (Abruzzo, Lazio, Marche, Tuscany, Umbria) and southern Italy (Basilicata, Calabria, Campania, Molise, Apulia, Sardinia, Sicily). The data collection form was developed in Microsoft Excel.

Specific IgG and IgM antibodies were detected with commercial automated tests CE marked in use at each laboratory and performed according to the manufacturers’ instructions. For *T. pallidum,* the number of positive or negative treponemal and non-treponemal tests were collected. Screening of possible syphilis cases is usually done by treponemal serological IgG immunoassay and/or *T. pallidum* particle agglutination (TPPA/TPHA). The non-treponemal rapid plasma reagine (RPR) flocculation test or venereal disease research laboratory (VDRL) are used to assess disease activity. For syphilis, positivity of any treponemal test with negative non-treponemal test is considered as past infection but in association with positive non-treponemal test as an active (non-latent) infection.

For each person, after removing any duplicates, only the first sample was considered. The number of acute infections per tested pathogen was reported by each laboratory. Acute infection for *T. gondii* and CMV was defined by presence of ascertained seroconversion (first test: IgM antibodies and no IgG antibodies and subsequent testing: IgG antibodies or IgM and IgG antibodies with previous negative results for both IgG and IgM antibodies) or presence of IgG and IgM antibodies with low or intermediate avidity index. A high IgG avidity index suggested a past infection [13,17].

For parvovirus B19, acute infections were defined as presence of IgM antibodies confirmed by detection of parvovirus DNA by commercial PCR tests.

For rubella, acute primary infections were defined as combined presence of IgM and ascertained IgG seroconversion and/or positive RT-PCR or low or intermediate IgG avidity index as recorded by the laboratories.

### Statistical analysis

Descriptive statistics were produced for each variable. Numbers and percentages are presented for categorical variables.

Meta-analytic techniques were used to obtain estimates; the pooled observed proportions per laboratory in each category were weighted according to the total number of samples processed via inverse variance weighting; binomial standard errors were calculated. Given the high heterogeneity in the analyses, restricted maximum likelihood was used for estimation. Heterogeneity was estimated with the I^2^, and Q statistics was used for homogeneity testing. Meta-regression was used to investigate factors associated with higher prevalence (or incidence). Laboratories with missing data on a pathogen and serological tests were given zero weight in the corresponding analysis (equivalent to not including them). The *meta* suite of commands in Stata version 16.0 (Stata Corporation, College Station, US) was used for statistical analysis.

## Results

Thirty-two laboratories from 19 of 20 Italian regions submitted pooled data on 685,211 samples from altogether 342,095 persons (Figure). No data were received from Molise region. All 32 laboratories submitted data on *T. gondii*, 23 on parvovirus B19, 28 on *T. pallidum* and 31 on rubella and 31 on CMV.

**Figure:**
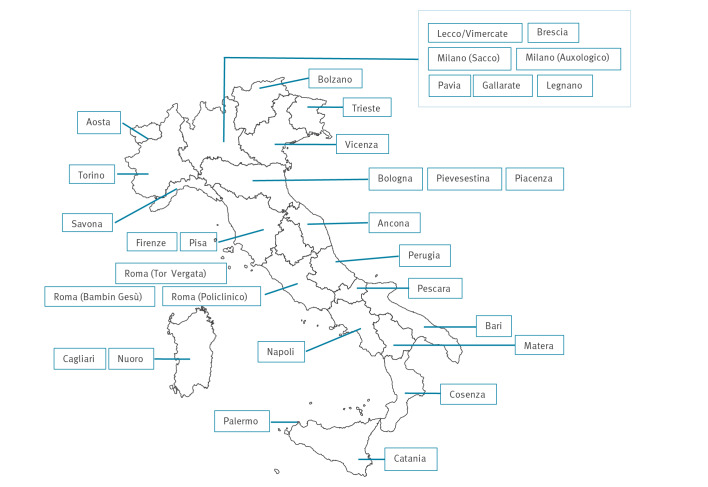
Distribution of laboratories participating in a study of antibodies against *Toxoplasma gondii*, parvovirus B19, *Treponema pallidum,* rubella virus and cytomegalovirus in women aged 16–45 years, Italy, 2019–2020 (n = 32)

### Toxoplasma gondii

Of the 114,678 women tested for antibodies against *T. gondii*, 13,700 (13%; 95% CI: 12–14) of 111,580 had IgG antibodies and 1,805 (3%; 95% CI: 1–4) of 114,678 had IgM antibodies (Table 1). Of the 1,805 women with IgM antibodies, 530 had acute infections (4/1,000 women tested; 95% CI: 3–4). Women with other nationalities had a higher proportion of IgG antibodies than those with Italian nationality (p < 0.001). No significant differences were observed between the included variables and IgM antibodies. Women with other nationalities had fewer acute infections than those with Italian nationality (p < 0.001) but women living in southern Italy had more acute infections than women in the other parts of the country (p = 0.039).

**Table 1:** Presence of IgG and IgM antibodies against *Toxoplasma gondii* and number of acute *Toxoplasma gondii* infections in women aged 16–45 years, Italy, 2019–2020 (n = 111,580)^a^

Characteristic	lgG antibodies					lgM antibodies					Acute infections^b^			
	Positive	Tested	%	95% CI	p value	Positive	Tested	%	95% CI	p value	n	Per 1,000	95% CI	p value
Nationality (n = 77,527)^c^														
Italian	5,446	57,324	10	9–11	Reference	1,156	58,643	3	1–6	Reference	369	5	4–7	Reference
Other	4,412	20,203	22	21–24	< 0.001	191	20,318	0	0–1	0.13	60	1	0–1	< 0.001
Total	9,858	77,527	15	14–17	NA	1,347	78,961	2	1–4	NA	429	2	2–3	NA
Age (years)														
16–25	1,643	13,105	13	11–14	Reference	253	13,425	1	1–2	Reference	79	2	1–3	Reference
26–35	7,019	61,931	12	11–14	0.18	907	63,571	2	1–3	0.85	302	7	4–10	0.16
36–45	5,038	36,544	14	13–15	< 0.001	645	37,682	4	1–9	0.31	149	4	3–5	0.68
Geographic area														
Northern Italy	10,316	86,662	13	12–14	Reference	1,119	88,349	3	0–6	Reference	259	3	2–4	Reference
Central Italy	2,267	18,177	12	10–14	0.04	287	19,343	1	1–2	0.44	109	5	3–4	0.91
Southern Italy	1,117	6,741	15	13–17	< 0.001	399	6,986	4	1–4	0.82	162	14	7–21	0.039
Total														
Participants	13,700	111,580	13	12–14	NA	1,805	114,678	3	1–4	NA	530	4	3–4	NA

CI: confidence interval; NA: not applicable.

^a^ Proportions were estimated by meta-analytical methods using weighting by the number of women tested in each participating laboratory.

^b^ Acute infections were defined as: IgG/IgM seroconversion and/or low or intermediate IgG avidity in IgM positive women.

^c^ Results from laboratories not submitting data by nationality were excluded from the analyses.

### Parvovirus B19

Of the 5,138 women tested for antibodies against parvovirus B19, 3,298 (65%; 95% CI: 60–69) had IgG antibodies and 205 (0%; 95% CI: 0–1) of 5,059 had IgM antibodies (Table 2). Thirty-three (16%) of the 205 women with IgM antibodies tested positive with PCR, confirming an acute infection (viral load > 10^4^ copies/mL). Twenty-seven of these women were Italian, six had other nationalities, four were aged 16–25 years, 17 were 26–35 years and 12 were 36–45 years. No significant differences were observed between Italian and other nationalities, ages or geographic area and presence of antibodies.

**Table 2:** Presence of parvovirus B19 IgG and IgM antibodies and number of acute parvovirus B19 infections in women aged 16–45 years, Italy, 2019–2020 (n = 5,138)^a^

Characteristic	lgG antibodies					lgM antibodies					Acute infections^b^			
	Positive	Tested	%	95% CI	p value	Positive	Tested	%	95% CI	p value	n	Per 1,000	95% CI	p value
Nationality (n = 3,814)^c^														
Italian	2,108	3,193	64	58–69	Reference	163	3,165	0	0–1	Reference	27	9	0–24	Reference
Other	353	621	57	47–65	0.15	31	598	4	1–9	0.45	6	4	0–32	0.66
Total	2,461	3,814	60	56–65	NA	194	3,763	3	1–5	NA	33	5	0–15	NA
Age (years)														
16–25	469	735	63	52–74	Reference	77	699	0	0–0	Reference	4	3	0–28	Reference
26–35	1,683	2,647	65	61–70	0.34	63	2,632	1	0–1	0.19	17	7	0–22	0.65
36–45	1,146	1,756	66	72–70	0.06	65	1,728	1	0–1	0.86	12	7	0–25	0.79
Geographic area														
Northern Italy	2,110	3,263	67	63–71	Reference	78	3,192	0	0–0	Reference	18	7	0–21	Reference
Central Italy	869	1,366	57	46–69	0.41	111	1,367	1	0–1	0.76	13	5	0–25	0.5
Southern Italy	319	509	70	72–70	0.57	16	500	1	0–1	0.55	2	2	0–32	0.48
Total														
Participants	3,298	5,138	65	60–69	NA	205	5,059	0	0–1	NA	33	6	0–17	NA

CI: confidence interval; NA: not applicable.

^a^ Proportions were estimated by meta-analytical methods using weighting by the number of women tested in each participating laboratory.

^b^ Acute infections were defined as: IgM antibodies and PCR positive (viral load > 10^4^).

^c^ Results from laboratories not submitting data by nationality were excluded from the analyses.

### Treponema pallidum

Of the 81,401 women tested for antibodies against *T. pallidum*, 889 (1%; 95% CI: 1–1) had IgG antibodies and 329 (0%; 95% CI: 0–1) had treponemal and non-treponemal antibodies (Table 3). The proportion of women with treponemal and non-treponemal antibodies was significantly higher in southern Italy than in the other parts of the country (p < 0.001).

**Table 3:** Presence of *Treponema pallidum* antibodies in women aged 16–45 years, Italy, 2019–2020 (n = 81,401)^a^

Characteristics	IgG antibodies					Treponemal and non-treponemal antibodies				
	Positive	Tested	%	95% CI	p value	Positive	%	95% CI	p value
Nationality (n = 55,546)^b^										
Italian	445	43,330	1	0–1	Reference	198	0	0–1	Reference
Other	134	15,329	1	1–1	0.19	89	0	0–1	0.006
Total	579	55,546	1	1–1	NA	287	0	0–1	NA
Age (years)										
16–25	106	10,890	0	0–0	Reference	65	0	0–0	Reference
26–35	413	43,870	1	1–2	0.001	161	1	1–2	0.72
36–45	370	26,641	1	1–2	0.003	103	1	1–2	0.77
Geographic area										
Northern Italy	530	67,165	1	0–1	Reference	132	1	0–1	Reference
Central Italy	107	9,115	1	0–1	0.045	47	4	0–1	0.07
Southern Italy	252	5,121	4	2–7	0.008	150	4	2–7	0.001
Total										
Participants	889	81,401	1	1–1	NA	329	0	0–1	NA

CI: confidence interval; NA: not applicable.

^a^ Proportions were estimated by meta-analytical methods using weighting by the number of women tested in each participating laboratory.

^b^ Results from laboratories not submitting data by nationality were excluded from the analyses.

### Rubella virus

Of the 69,865 women tested for antibodies against rubella virus, 63,828 (87%; 95% CI: 85–88) had IgG antibodies and 2,998 (2%; 95% CI: 1–3) of 55,327 had IgM antibodies (Table 4). The proportion of women with IgG antibodies was lower in southern Italy than in the other parts of the country (p < 0.001). No acute rubella cases were diagnosed.

**Table 4:** Presence of IgG and IgM antibodies against rubella virus in women aged 16–45 years, Italy, 2019–2020 (n = 69,865)^a^

Characteristics	lgG antibodies					lgM antibodies				
	Positive	Tested	%	95% CI	p value	Positive	Tested	%	95% CI	p value
Nationality (n = 48,908)^b^										
Italian	33,661	36,818	86	84–88	Reference	438	27,891	1	1–1	Reference
Other	10,865	12,090	86	84–88	0.35	146	12,027	2	1–5	0.55
Total	44,526	48,908	86	85–88	NA	584	39,918	2	1–5	NA
Age (years)										
16–25	11,028	12,127	85	83–87	Reference	110	7,404	0	0–0	Reference
26–35	34,281	37,603	86	83–88	0.25	2,639	30,821	3	1–7	0.42
36–45	18,519	20,135	89	87–91	0.003	249	17,102	1	1–1	0.39
Geographic area										
Northern Italy	48,820	52,828	90	89–91	Reference	2,708	42,902	2	0–5	Reference
Central Italy	12,297	13,590	87	85–88	0.09	158	9,166	1	0–1	0.82
Southern Italy	2,711	3,447	77	74–80	< 0.001	132	3,259	2	1–3	0.11
Total										
Participants	63,828	69,865	87	85–88	NA	2,998	55,327	2	1–3	NA

CI: confidence interval; NA: not applicable.

^a^ Proportions were estimated by meta-analytical methods using weighting by the number of women tested in each participating laboratory.

^b^ Results from laboratories not submitting data by nationality were excluded from the analyses.

### Cytomegalovirus

Of the 71,013 women tested for antibodies against CMV, 45,558 (66%; 95% CI: 64–68) had IgG antibodies and 3,452 (3%; 95% CI: 2–3) of 69,968 had IgM antibodies (Table 5). We estimated 449 acute infections (4/1,000; 95% CI: 3–5). Women with other nationalities had a higher proportion of IgG antibodies but a lower proportion for IgM antibodies and fewer acute infections than those with Italian nationalities (p < 0.001). Women living in southern Italy had higher proportions of IgG and IgM antibodies than women living in the other parts of the country (p < 0.001).

**Table 5:** Presence of IgG and IgM antibodies against cytomegalovirus and number of acute cytomegalovirus infections in women aged 16–45 years, Italy, 2019–2020 (n = 71,013)^a^

Characteristic	lgG antibodies					lgM antibodies					Acute infections^b^			
	Positive	Tested	%	95% CI	p value	Positive	Tested	%	95% CI	p value	Number	Per 1,000	95% CI	p value
Nationality (n = 50,361)^c^														
Italian	21,087	36,280	59	56–61	Reference	2,441	35,640	4	3–4	Reference	358	8	6–10	Reference
Other	12,088	14,081	93	91–95	< 0.001	406	13,827	1	0–1	< 0.001	18	0	0–1	< 0.001
Total	33,175	50,361	75	72–78	NA	2,847	49,467	2	2–3	NA	379	3	2–3	NA
Age groups (years)														
16–25	6,456	9,813	66	63–69	Reference	616	9,760	3	2–4	Reference	78	4	2–6	Reference
26–35	23,304	36,688	65	62–69	0.54	1,765	37,088	3	2–4	0.6	252	6	4–8	0.68
36–45	15,798	24,512	66	64–69	0.31	1,071	23,120	2	2–3	0.37	119	4	3–5	0.56
Geographic area														
Northern Italy	33,460	53,454	64	62–67	Reference	2,466	52,143	2	2–2	Reference	226	3	2–4	Reference
Central Italy	8,553	12,414	67	63–71	0.06	581	12,679	3	2–4	0.09	117	7	4–9	0.41
Southern Italy	3,545	5,145	68	65–71	<0.001	405	5,146	6	4–7	< 0.001	106	13	6–19	0.32
Total														
Participants	45,558	71,013	66	64–68	NA	3,452	69,968	3	2–3	NA	449	4	3–5	NA

CI: confidence interval; NA: not applicable.

^a^ Proportions were estimated by meta-analytical methods using weighting by the number of women tested in each participating laboratory.

^b^ Acute infections were defined as: IgG/IgM seroconversion and/or low or intermediate IgG avidity in IgM positive women.

^c^ Results from laboratories not submitting data by nationality were excluded from the analyses.

## Discussion

In Europe, data on the prevalence of TORCH agents in women of fertile age are sparse. We estimated presence of antibodies against five pathogens in women aged 16–45 years in Italy. Furthermore, we investigated associations between proportions of antibodies and age and geographic area. As we received data during a 12-month period from 32 laboratories from all Italian regions but one, including all the major referral centres for antenatal screening of TORCH infections, the study results can be considered representative of the status in women of fertile age accessing healthcare as part of routine practice. The results highlight that fertile women might benefit from specific advice on preventive measures and closer clinical follow-up.

The proportion of IgG antibodies against *T. gondii* was 13%, similar to some other studies. Seroprevalence of *T. gondii* antibodies has decreased in Italy [18] and some other countries [19,20]. As *T. gondii* is often transmitted via food, the decrease could be due to changes in alimentary habits, farming systems and improved hygienic practices [19]. The presence of IgG antibodies was significantly higher among women with other nationalities than the Italian. Seroprevalence increase with increasing age has been previously reported [21,22] and could be due to multiple exposures to *T. gondii* throughout life. Presence of IgM antibodies against *T. gondii* was less common (3%). However, testing for IgM antibodies is not sufficient to define an acute infection [23]. In our study, 29.3% of women with IgM antibodies (4/1,000; 95% CI: 3–4) had acute infections as assessed by IgG and IgM seroconversion and low or intermediate avidity. Toxoplasmosis can have a high disease burden and treatment of gestational toxoplasmosis improves the prognosis. Thus, also with a low seroprevalence, screening will be cost-effective [24,25]. As *T. gondii* is endemic in wild and domestic animals in Italy [16], it is important to maintain the screening programme as pointed out in the Italian Guidelines for Physiological Pregnancy [10].

Of the 5,138 women tested, 65% had IgG antibodies against parvovirus B19, with no statistically significant differences between nationality, age and geographic area, contrary to a recent study in Italy [11]. Of the 5,059 women tested, 205 (2%) had IgM antibodies and 33 had acute infections. Even if serological screening is not recommended in pregnancy, during parvovirus B19 outbreaks when seroconversion rates are higher, assessment for maternal immunity is relevant to identify women at risk of acquiring B19 infection in pregnancy.

In 2016, WHO launched the 2016–2021strategy of global curable and incurable STIs and included *T. pallidum* among infections that need immediate action*.* In 2019, *T. pallidum* was included in testing of women of fertile age in Italy. In our study, 889 women had anti-*T. pallidum* antibodies and 329 were positive in treponemal and non-treponemal tests. The proportion of IgG antibodies was higher among women in southern Italy, confirming results from a previous study [15]. The Italian guidelines recommend serological screening of all pregnant women for syphilis in the first and the third trimester, which we support. To confirm the diagnosis, seropositive women need to be tested with TPHA and RPR or VDRL test [10].

According to data from the Italian Ministry of Health, in 2020, vaccination coverage against rubella virus was around 90% [15]. In our study, the overall proportion of IgG antibody positivity was 87% and significantly lower in southern Italy (77%). In previous studies, the seroprevalence was around 90% in northern Italy, 85% in central Italy and 81% in southern Italy [15,26,27]. This could reflect a lower vaccination coverage in southern Italy. We observed a significantly higher proportion of IgG antibody positivity among women aged 36–45 years. Differences across age groups have been seen in other studies [27]. Measurement of IgG antibodies against rubella virus with some of the assays used in our study may have led to false-negative results, potentially triggering unnecessary booster vaccinations. However, the number of notified rubella cases has not increased, and no congenital rubella syndrome cases have been recently reported in Italy [28]. Of the tested women in our study, 2% had IgM positive results for rubella antibodies but no acute infections were observed. Test results for IgM antibodies can be false-positive or caused by persistent IgM reactivity after infection or vaccination [27]. Women should be informed that rubella-specific IgM positive results without clinical symptoms in a previously vaccinated person are most likely not an indication of an acute infection: further testing may not be warranted and termination of pregnancy unnecessary.

The current Italian guidelines do not recommend testing for rubella antibodies in pregnancy [10]. However, European Centre for Disease Prevention and Control (ECDC) reported 227 cases of rubella in 2024. As the estimated proportion of IgG seropositivity in southern Italy was below 85% in our study, in the absence of measles–mumps–rubella (MMR) vaccination certification, it would seem prudent to test women of fertile age for rubella IgG antibodies to assess immunity to the virus and take actions to prevent congenital infection [28,29].

Of the tested women, 66% had IgG antibodies against CMV. The lower the seroprevalence of CMV, the higher the risk of acute infection during pregnancy [30]. A total of 3,452 (3%) women had IgM antibodies against CMV, and IgM antibodies can be associated with both primary and secondary CMV infections. We identified 449 acute infections (4/1,000; CI 95%: 3–5), with no differences between the three age groups. Presence of antibodies was lower among women with Italian nationality than among other nationalities. Exposure to CMV may be associated with education level, social status and lifestyle, population density, number of children per family and child-rearing practices [31]. Interventions to reduce the risk of maternal CMV infection are limited to behavioural practices. Valacyclovir has been recently approved to prevent vertical transmission. Thus, our comprehensive study on CMV infection in women of reproductive age is relevant towards our understanding of CMV and associated disease, which can guide public health strategies. Our study results support and confirm the national strategy of conducting screening for CMV in pregnancy [10].

We acknowledge some limitations. Only aggregated data were used in the analyses; therefore, we have been able to study only some factors, such as age group, nationality and geographic area. Also, the participating laboratories used a variety of commercially available tests, and we could not take into account the potential variability in test performances. Finally, the concomitant COVID 19 pandemic affected participation of the laboratories, collection and analysis of datasets.

## Conclusion

Presence of IgG and IgM antibodies against TORCH pathogens *T. gondii*, parvovirus B19, *T. pallidum*, rubella virus and CMV in women of fertile age in Italy varied. As *T. gondii* and *T. pallidum* infections are a risk for the fetus, it is important to maintain the screening programme. Assessment for maternal immunity to parvovirus B19 in pregnancy is relevant to identify women at risk, especially during outbreaks. It is still important to test women of fertile age for immunity against rubella virus and CMV to prevent congenital infection. In fertile age, the identification of IgG seronegative women and an intervention based on vaccination for rubella virus before pregnancy and hygiene recommendations for CMV in pregnancy have the potential to prevent maternal and congenital infection. Our study can provide the rationale to improve existing prevention strategies in Italy.

## Data Availability

The dataset is available from the corresponding author upon reasonable request. A Data Transfer Agreement will be a requirement.
